# Non-radiographic methods of measuring global sagittal balance: a systematic review

**DOI:** 10.1186/s13013-017-0135-x

**Published:** 2017-10-03

**Authors:** Larry Cohen, Sarah Kobayashi, Milena Simic, Sarah Dennis, Kathryn Refshauge, Evangelos Pappas

**Affiliations:** Faculty of Health Sciences, Discipline of Physiotherapy, The University of Sydney, 75 East Street, Lidcombe, NSW 2141 Australia

**Keywords:** Spine posture, Spine shape, Non-invasive assessment, Sagittal vertical axis, SVA, Measurement, Reliability, Validity

## Abstract

**Background:**

Global sagittal balance, describing the vertical alignment of the spine, is an important factor in the non-operative and operative management of back pain. However, the typical gold standard method of assessment, radiography, requires exposure to radiation and increased cost, making it unsuitable for repeated use. Non-radiologic methods of assessment are available, but their reliability and validity in the current literature have not been systematically assessed. Therefore, the aim of this systematic review was to synthesise and evaluate the reliability and validity of non-radiographic methods of assessing global sagittal balance.

**Methods:**

Five electronic databases were searched and methodology evaluated by two independent reviewers using the13-item, reliability and validity, Brink and Louw critical appraisal tool.

**Results:**

Fourteen articles describing six methodologies were identified from 3940 records. The six non-radiographic methodologies were biophotogrammetry, plumbline, surface topography, infra-red motion analysis, spinal mouse and ultrasound. Construct validity was evaluated for surface topography (*R* = 0.49 and *R* = 0.68, *p* < 0.001), infra-red motion-analysis (ICC = 0.81) and plumbline testing (ICC = 0.83). Reliability ranged from moderate (ICC = 0.67) for spinal mouse to very high for surface topography (Cronbach α = 0.985). Measures of agreement ranged from 0.9 mm (plumbline) to 22.94 mm (infra-red motion-analysis). Variability in study populations, reporting parameters and statistics prevented a meta-analysis.

**Conclusions:**

The reliability and validity of the non-radiographic methods of measuring global sagittal balance was reported within 14 identified articles. Based on this limited evidence, non-radiographic methods appear to have moderate to very high reliability and limited to three methodologies, moderate to high validity. The overall quality and methodological approaches of the included articles were highly variable. Further research should focus on the validity of non-radiographic methods with a greater adherence to reporting actual and clinically relevant measures of agreement.

## Background

Progressive stooped posture, a common consequence of the ageing process, is associated with poor quality of life [1, 2]. This posture, which can be described according to the vertical alignment of the trunk over the pelvis, is defined as global sagittal balance and is termed anterior sagittal balance when exceeding predetermined threshold values. Anterior sagittal balance is the postural deformity that is most closely correlated with pain, activity limitations and reduced quality of life [2] and affects up to 29% of the population above 60 years of age [3].

The current gold standard for measurement of global sagittal balance is the sagittal vertical axis (SVA) obtained via radiographs. SVA is quantified by measuring, in centimetres, the horizontal distance between the centre of the C7 vertebral body to the postero-superior border of the sacrum on full-length lateral spine radiographs [1]. This requires the use of spine-specific radiographic software [4] which demonstrates excellent intra-rater (ICC = 0.98) and inter-rater (ICC = 0.95) reliability and excellent accuracy between inter-rater tests (ISO reproducibility of 4.02 mm) [5]. SVA thresholds defining anterior sagittal balance range from 3 to 6 cm [6–10]. Alternate radiographic methods of sagittal spine balance measurement, which do not require spine specific radiographic software, include the angular measurements of T1 spinal inclination (T1Spi) and C7-S1 trunk inclination [11]. T1Spi has been reported to be more closely correlated to clinical outcomes evaluated by the Oswestry Disability Index, Short Form-12 and SRS-23 than SVA [11].

Recent advances in surgical and non-surgical spine management have revealed the importance of identifying, maintaining or restoring sagittal balance to achieve reduction in pain, improvement in function, quality of life and reduction in post-operative complications following spine surgery [11, 12]. Physiotherapy treatment aimed at restoring sagittal balance, primarily by increasing lumbar lordosis, has likewise been demonstrated to improve clinical outcomes in patients with chronic lower back pain [13]. Therefore, the measurement of global sagittal balance is important for the development and monitoring of effective spine therapy interventions.

Although radiographs are the current gold standard, repeated radiographic exposure potentially increases lifetime risk for cancer development [13]. This is compounded when considering that lateral full spine radiographs can deliver an effective radiation dose that is 50–70% higher than standard posterior-anterior (PA) full spine radiographs [14]. Therefore, due to the high cost and radiation exposure, repeated radiographic measurement and monitoring of sagittal balance in the clinical setting have serious limitations [13]. Non-radiographic methods of measuring global sagittal balance are available and may present a viable option for monitoring patient progress. These methods vary with regard to technical complexity and equipment cost. However, the currently available methods and their psychometric properties have not been assessed systematically. Therefore, the aim of this systematic review was to evaluate the reliability and validity of non-radiographic methods of assessing global sagittal balance.

## Methods

### Protocol and registration

This review protocol was registered in August 2014 with the PROSPERO International prospective register of systematic reviews (ID PROSPERO 2014:CRD42014013071).

### Data sources

Electronic database searches of MEDLINE, EMBASE, Web of Science, CINAHL and AMED were conducted from database inception until week 38, September 2016. The search terms were based on three main term groups: sagittal alignment, psychometric properties and physical tests.

The Boolean term “OR” was used within each term group and the Boolean term “AND” was used between each term group. Additional hand searches of relevant bibliographies were completed (Appendix).

### Eligibility criteria

Studies were included if they reported reliability and/or validity of non-radiographic methods of measuring standing global sagittal spine parameters in people with or without spine deformity or pain. All studies were considered regardless of publication date, age of participants or language.

### Study selection

Two independent reviewers (LC, SK), after trialling a small pilot study, screened the titles and abstracts for eligible studies and reviewed the full texts of those identified. Full texts were retrieved if one reviewer determined that the record could not be excluded by title or abstract. In cases of disagreement, a third reviewer (EP) adjudicated. Bibliographies of included studies were searched for additional references.

### Data extraction

In order to extract comprehensive methodological, population and psychometric data two independent reviewers (LC, SK) used a 13-item critical appraisal tool developed by Brink and Louw [15]. The Brink and Louw critical appraisal tool was developed from the Quality Assessment of Diagnostic Accuracy Studies (QUADAS) and Quality Appraisal of Diagnostic Reliability Studies (QUAREL) to test combined or independent reliability and validation studies [16]. The data included a description of the study population and raters, detailed description of blinding, randomisation, between testing time periods, testing procedures, withdrawals and statistics methodology. Disagreement was resolved by consensus and, if necessary, in consultation with a third reviewer (EP). Authors of articles where the results or methodology were unclear were contacted for clarification.

Pearson’s *r*, Cronbach α and intra-class correlation coefficients (ICC) statistics were interpreted as follows: ≤ 0.29 very low correlation, 0.20–0.49 low correlation, 0.50–0.69 moderate correlation, 0.70–0.89 high correlation and ≥ 0.90 very high correlation [17]. Agreement was evaluated by the standard error of measurement (SEM) which, when data were available, was calculated according to the equation: $\mathrm{SEM}=\mathrm{standard}\  \mathrm{deviation}\ \left(\mathrm{SD}\right)\div \sqrt{1}-\mathrm{reliability}\  \mathrm{coefficient}$ [18].

### Quality assessment

Methodological quality of individual studies was evaluated using the Brink and Louw critical appraisal tool and synthesised within the summary tables. Articles were considered high quality if they scored greater than the accepted 60% threshold on the Brink and Louw critical appraisal tool [16].

## Results

### Studies included in the review

**Fig. 1:**
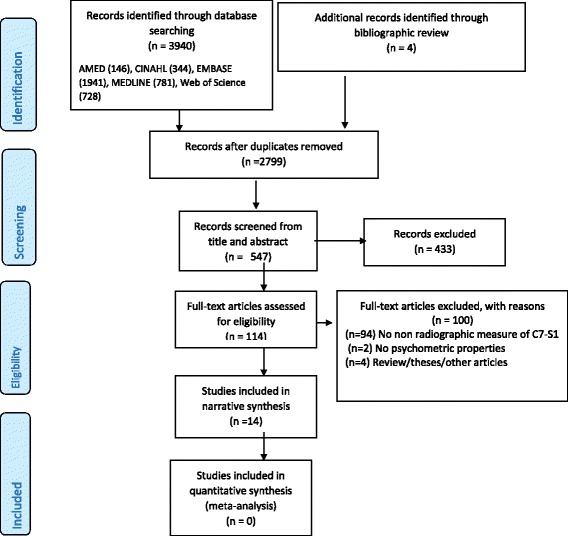
PRISMA flow diagram describing selection process for included studies

The database search strategy retrieved a total of 3940 records. After removal of duplicates, 2685 of the remaining citations were excluded as they did not meet the inclusion criteria. Following full text review of 114 articles, 14 articles met the inclusion criteria. The flow of articles through the review process is depicted in the PRISMA flow diagram (Fig. 1). We contacted the lead author of three included studies, a German language article for further information on methodology [19] and the lead authors of two other English language studies, to clarify reported units of measurement [20] and methods of measurement [21].

### Global sagittal balance measurement methods

A total of 14 studies describing six global sagittal balance measurement methods were included in the review. Two studies measured construct validity, one by root mean square deviation [19] and one by ICC [21], two measured both construct validity and reliability [13, 22] and 10 studies [20, 23–31] investigated reliability of the sagittal balance measurement methods.

A description of each non-radiographic measurement method is provided in Table 1. Of the four studies reporting validity, three studies compared surface topography to radiographically measured angular trunk inclination [13, 22] and radiographic SVA [19]. The fourth validity study compared plumbline and infra-red (IR) motion analysis to radiographic SVA [21]. Nine studies examined inter- and intra-rater reliability [13, 19, 20, 22–25, 29, 31], and three studies examined test-retest time interval reliability [26–28]. Five studies evaluated the reliability for surface topography and two studies each for spinal mouse, plumbline testing and biophotogrammetry with one study for ultrasonic testing.

**Table 1:** Detailed description of non-radiographic measurement methods, equipment and technique used in the included studies

Method	Description of evaluation	Equipment required	Technique	References
Biophotogrammetry	Biophotogrammetric analysis involves measuring, off-lateral posture photographs, the distance from a plumbline to the lordotic and cervical apex [25] or C7, S1 prominences [30].	Digital camera with vertical plumbline reference posterior to the subject within field of view and a known (presized) object within field of view to establish distance scaling. Computer with graphic editing software	Adhesive stickers that can be seen from the lateral margin of the body are placed on the C7 and S1 landmarks. After calibration, the distance from the plumbline to the landmark points are measured using graphic editing software.	[25, 30]
Infra-red motion analysis	Motion analysis computer-interfaced stereovideographic acquisition of infra-red-activated anatomical markers at C7 [21, 26], T1 [28] and S1.	Minimum of three motion analysis cameras linked to a computer via an image processor. Infra-red light reflected on the adhesive markers	Adhesive infra-red markers are affixed to C7/T1 and S1. The markers are activated by infra-red light and the dedicated computer system triangulates the spine data measuring the sagittal arrows.	[21, 26, 28]
Plumbline	A ruler and plumbline to measure the distance to the C7 and L3 [29, 31], or C7 and S1 [21] anatomical points on the body	Ruler and plumbline	The plumbline is held against or very near to the posterior surface of the skin. The distance from the plumbline to C7 and L3 or S1 is measured.	[21, 29, 31]
Spinal mouse	Spinal mouse assessment uses a wireless computer-interfaced rollerball input device to determine the inclination of the spine from C7 to S1 and the vertical.	Spinal Mouse (Idiag, Voletswil, Switzerland) and computer	The spinal mouse is rolled along the contour of the spine from C7 to S1 measuring distance of travel and angulation.	[23, 24]
Surface topography	Surface topography based on Moire stereovideography measures the distortion of a predicted light grid to create a 3D model of the back providing angular or distance offset data from the vertebral prominens (C7 or T1) to the midpoint between the PSIS.	Surface topography machine (Biomod, AXS Ingenierie, Bordeaux, France) [13] formetric (Diers International, Schlangenbad, Germany) [19, 20, 22, 27] and computer interface	Depending on system, optional, infra-red adhesive markers are placed on C7, PSISs and inter-gluteal cleft. Scanning is performed according to the specifications of the manufacturer.	[13, 19, 20, 22, 27]
Freepoint ultrasound	Freepoint ultrasound system emits an ultrasonic signal from the probe to receivers which triangulate the position of T1 and C7 in space.	Freepoint ultrasound system (GTCO Calcomp, Scottsdale, USA) and interfaced computer	The freepoint probe is used to identify the T1 and S1 landmarks, which are triangulated and digitised allowing for computerised 3D reconstruction.	[28]

In terms of the outcome variables, trunk inclination was measured in four studies; two using spinal mouse [23, 24] and two using surface topography [13, 22] methodology. The distance from a plumbline reference line to the cervical or lumbar lordosis apex and the S1 landmark point was measured in four studies [13, 21, 25, 29]. These plumbline reference line-to-body surface landmark points are commonly termed “sagittal arrows” in the literature [21]. The horizontal offset between superior and inferior landmarks was measured in seven studies, but there was inconsistency with landmark identification. Three studies used the vertebra prominens and the midpoint of the lumbar dimples [19, 20, 27], one study C7 and the midpoint of the lumbar dimples [21], two studies used C7-S1 [26, 30], and one study used T1-S1 [28].

### Quality assessment

The average quality of the 14 studies was 56% (range 44–77%) (Table 2). One validity and reliability study [22], two validity studies [19, 21] and three reliability studies [23, 25, 27] were of high quality, scoring > 60% on the critical appraisal tool. The main items with low scores were a suitable description of the raters (71% of studies unreported), within-rater blinding (77% of studies unreported), variation of testing order between raters (92% of studies unreported) and a suitable explanation of withdrawals from the study (92% of studies unreported).

**Table 2:** Methodological quality of included studies evaluated using the Brink and Louw critical appraisal tool

Study	Key information	1	2	3	4	5	6	7	8	9	10	11	12	13	High-quality > 60%
1	de Seze [21]	✓	✗	✓	n/a	n/a	n/a	✓	n/a	✓	✗	✓	✗	✓	6/9 = 66%
2	Grosso 2002 [31]	✓	✓	n/a	✗	✗	✗	n/a	✓	n/a	✗	n/a	✗	✓	4/9 = 44%
3	Kellis 2008 [23]	✓	✓	n/a	✓	✓	✗	n/a	✓	n/a	✓	n/a	✗	✓	7/9 = 77%
4	Knott 2016 [22]	✓	✗	✓	✗	✗	✗	✓	✓	✓	✓	✓	✗	✓	8/13 = 62%
5	Legaye 2012 [13]	✓	✗	✓	✗	✗	✗	✗	✗	✓	✓	✓	✗	✓	6/13 = 46%
6	Liljenqvist 1998 [19]	✓	✗	✓	n/a	n/a	n/a	✗	✗	✓	✓	✓	✗	✓	6/9 = 66%
7	Mannion 2004 [24]	✓	✗	n/a	✓	✗	✗	n/a	✗	n/a	✓	n/a	✗	✓	4/9 = 44%
8	Mohokum 2010 [20]	✓	✓	n/a	✗	✗	✗	n/a	✓	n/a	✓	n/a	✗	✓	5/9 = 55%
9	Milanesi 2011 [25]	✓	✗	n/a	✓	✓	✗	n/a	✓	n/a	✓	n/a	✗	✓	6/9 = 66%
10	Negrini 2001 [26]	✓	✗	n/a	✗	✗	✗	n/a	✓	n/a	✓	n/a	✓	✓	5/9 = 55%
11	Schroeder [27]	✓	✓	n/a	✗	✓	✗	n/a	✓	n/a	✓	n/a	✗	✓	6/9 = 66%
12	Zabjek 1999 [28]	✓	✗	n/a	✗	✗	✓	n/a	✓	n/a	✓	n/a	✗	✓	5/9 = 55%
13	Zaina 2012 [29]	✗	✗	n/a	✗	✗	✗	n/a	✓	n/a	✓	n/a	✗	✓	4/9 = 44%
14	Zheng 2010 [30]	✓	✗	n/a	✗	✗	✗	n/a	✓	n/a	✓	n/a	✗	✓	4/9 = 44%

*1* description of study population, *2* description of raters, *3* explanation of reference standards (validity only), *4* between rater blinding (reliability only), *5* within rater blinding (reliability), *6* variation of testing order (reliability), *7* time period between index test and reference standard (validity), *8* time period between repeated measures (reliability), *9* independency of reference standard from index test (validity), *10* description of index test procedure, *11* description of reference test procedure (validity), *12* explanation of any withdrawals, *13* appropriate statistics methods. ✓ Reported, ✗ Not reported

### Participants

Healthy adult participants were evaluated in five studies [20, 24, 27, 28, 30] and healthy children in one study [23]. Four studies evaluated participants with spine deformity or pain; three included adolescents [22, 26, 31] and one involved adults [13]. One study evaluated children, adolescents and adults with spine deformity [19], one study evaluated adults who demonstrated clinical manifestation of mouth breathing during childhood [25] and another study, adults with camptocormia [21].

Sample sizes for the validity studies ranged from 95 [19] to 326 [13] participants for the two surface topography studies and 49 participants for the plumbline and IR motion study [21]. Reliability study sample sizes ranged from two participants examined once by five raters (inter-rater) and 15 times by one rater (intra-rater) [13] to 180 participants examined by two raters (inter-rater) and then repeated after 5 min by one rater (intra-rater) [29]. Only four studies included participants with a mean age greater than 30 years [13, 21, 24, 30].

### Validity and reliability

#### Validity

Correlations between non-radiographic and radiographic methods of measuring global sagittal balance ranged from low to high (Table 3). Liljenquist et al. [19] compared surface topography sagittal trunk offset distance to radiographic SVA and reported a root mean square deviation (RMSD) of 1.07 cm. Legaye [13] compared surface topography trunk inclination to radiographically determined C7-S1 global sagittal axis and reported a moderate and significant correlation of *r* = 0.68 (*p* < 0.001). Knott et al. [22] compared surface topography sagittal trunk inclination to radiographically determined SVA inclination and reported a low Pearson correlation of 0.49. de Seze et al. [21] compared radiographic SVA to plumbline and IR motion analysis and reported high ICCs of 0.81 and 0.83 respectively.

**Table 3:** Study characteristics, reliability, validity and SEM data of included studies

Non-radiographic method	Study	Index test variable	Sample	Age	Methodology description	Validity test variable	Reliability test variable	Statistical measure	Resultant statistical value	SEM
Biophotogrammetric analysis	Milanesi 2011 [25]	Cervical and lumbar lordosis apex arrows	24 adults with clinical manifestation of mouth breathing during childhood	18–30 years	3 raters on 1 occasion		Inter-rater	ICC	> 0.75	0.23−0.37 cm (range)
	Zheng 2010 [30]	C7-S1 offset	30 asymptomatic adult participants	35.5 ± 9.4 years	Examined 12 times in neutral standing and hands on clavicles		Intra-rater	Repeatability (mean of the SD ± SD)		6 ± 1.9 mm neutral standing
								As above		7.3 ± 3 mm hands on clavicles
Freepoint (FP) ultrasound system	Zabjeck 1999 [28]	T1-S1 offset	15 adult control participants	25 ± 6 years	Examined 5 times by each system 1 week apart		FP intra-session	Mean ± SD	19.1 ± 7.9 mm	2.03 mm (mean)
							FP inter-session difference	Mean ± SD	−3.2 ± 11.6 mm	2.99 mm (mean)
							MA vs. freepoint	ICC	0.93	
Infra-red motion analysis	de Seze 2015 [21] Elite IR optoelectronic system	C7-S1 offset	43 adults with camptocormia	69 ± 10 years		Validity. Radiographic sagittal vertical axis (SVA)		ICC	0.83	
	Negrini 2001. [26] Auscan optoelectronic 3D IR imaging system with manual landmark identification	C7-S1 offset	97 patients with adolescent idiopathic scoliosis	15.15 ± 2.25 years	Examined twice with 3 time intervals between measurements		Intra-session 6 s interval	Bland and Altman repeatability coefficient		12.52 mm (mean difference)
							Intra-session 24 s interval	As above		14.64 mm (as above)
							Intra-session 167 s interval	As above		22.94 mm (as above)
	Zabjeck 1999 [28] IR motion analysis (MA) system and freepoint (FP) ultrasound system	T1-S1 offset	15 adult control participants	25 ± 6 years	Examined 5 times by each system 1 week apart		MA intra-session difference	Mean ± SD	10.9 ± 7 mm	1.8 mm (mean)
							MA inter-session difference	Mean ± SD	2.9 ± 6.9 mm	1.78 mm (mean)
Plumbline testing	de Seze 2015 [21]	C7-S1 Sagittal arrows	43 adults with camptocormia	69 ± 10 years		Validity. Radiographic sagittal vertical axis (SVA)		ICC	0.81	
	Grosso 2002 [31]	C7-L3 sagittal arrows	116 AIS, hyperkyphotic and hyperlordotic adolescents	13.6 ± 2.4 years	2 raters on 2 occasions		Inter-rater	ICC cervical	0.86	
								ICC lumbar	0.76	
	Zaina 2012 [29]	C7 and L3 Sagittal arrows	180 AIS and hyperkyphotic adolescents	Aged 11–16	Examined by 2 raters and then repeated after 5 min by one rater		Intra-rater	Bland and Altman repeatability coefficient		0.9 mm C7 1.2 mm L3 (mean difference)
							Inter-rater	As above		1.7 mm C7 2.2 mm L3
Spinal mouse	Kellis 2008 [23]	C7-S1 Angular trunk inclination	81 healthy children	10.6 ± 1.7 years	Examined by 3 raters on 2 separate occasions		Intra-rater	ICC	0.67–0.87	1.19°–1.97° (range)
							Inter-rater	ICC	0.77–0.82	0.96°–1.2°
	Mannion 2004 [24]	C7-S1 Angular trunk inclination	29 healthy adult participants	45.4 ± 7.7 years	Examined by 2 raters on 2 separate occasions		Intra-rater	ICC	0.83–0.84	1° (0.8°–1.5)° (mean)(95% CI)
							Inter-rater	ICC	0.71–0.77	1.5° (1.2–2.2 95% CI) (as above)
Surface topography	Knott 2016 [22] Diers formetric surface topography system compared with upright full spine radiographs	VP-DM sagittal trunk inclincation. Compared with C7-S1 trunk inclination	193 AIS and hyperkyphotic adolescents	8–18 years	Multicentre trial with same day testing.	Validity. Radiographic sagittal vertical inclination		Pearson’s Correlation	0.49	± 3.7° (SD)
							Three scans repeated within 5 min	ICC	0.91	± 1.1° (SD)
	Legaye 2012 [13]. Biomod surface topographical system with manual landmark identification	C7 and superior border of gluteal cleft angular trunk inclination	1 symptomatic male, 1 asymptomatic scoliotic female participant	Both 53-year olds	Examined once by 5 raters (inter-observer) and 15 times by one rater (intra-observer).		Intra-rater	Confidence interval	1°	
							Inter-rater	Confidence interval	1°	
		C7 and superior border of -gluteal cleft (pelvic) sagittal arrows			As above		Intra-rater	Confidence interval	3 mm cervical	
								Confidence interval	5 mm pelvic	
							Inter-rater	Confidence interval	4 mm cervical	
								Confidence interval	4 mm pelvic	
		C7 and superior border of -gluteal cleft Angular trunk inclination	326 adults with pain or deformity(kyphosis, fractures, scoliosis)	Range from 7 to 86 years	Correlation between radiographs and surface topography	Validity. Radiographic C7S1 angular axis		Pearson’s correlation	*R* = 0.68 *p* < 0.001	
	Liljenqvist 1998 [19] Diers formetric surface topography system compared with upright full spine radiographs	VP-DM sagittal offset distance	95 children, adolescents and adult patients with scoliosis or hyperkyphosis	Mean age 16.5 range 7–30 years	Correlation between radiographs and surface topography examined by 2 raters	Validity. Radiographic sagittal vertical axis (SVA)		Root mean square deviation	1.07 cm	
	Mohokum [20] 2010Diers formetric surface topography system with automatic landmark identification	VP-DM sagittal offset distance^a^	51 healthy adults with normal and high BMI	24.6 ± 5.8 years	Examined 3 times by 3 raters on one occasion					3.49 mm (mean)
							Intra-rater	Cronbach α	0.950–0.985	
							Inter-rater	Cronbach α	0.97	
	Schroeder [27] 2015Diers formetric surface topography system with automatic landmark identification	VP-DM sagittal offset distance	20 adult participants without back pain	25.4 ± 5.5 years	Within 5 min on 1 day, the following day and the following week					3 mm (mean)
							Intra-day	ICC	0.858–0.978	
							Inter-day	ICC	0.843–0.977	
							Inter-week	ICC	0.855–0.977	

^a^Erroneously reported as degrees

*VP* vertebra prominens, *DM* midpoint between *PSIS* dimples, *SEM* standard error of measurement

#### Reliability

The overall reliability results of all non-radiographic measurements ranged from moderate (ICC 0.67) to very high (Cronbach α 0.98). Spinal mouse methodology rated moderate (ICC 0.67) to high (ICC 0.87) [23, 24], biophotogrammetric (ICC > 0.75) [25] and plumbline measurement (ICC 0.76–0.86) [31] rated high, and surface topography inter- and intra-rater reliability rated high (ICC 0.84) [27] to very high (Cronbach α 0.95) [20]. The repeatability coefficient of the three methods reporting reliability by Bland and Altman statistics ranged from 0.9 mm [29] to 22.9 mm [32]. The results of the descriptive statistics depicting the reliability of the remaining three methods ranged from 3 mm [13] to 19.1 mm [28]. The test-retest order of precision from most to least precise was plumbline (0.9–1.2 mm) [29], surface topography (3–5 mm) [13], bio-photogrammetry (6–7.3 mm) [30], motion analysis (2.9–10.9 mm) [28], freepoint ultrasound (3.2–19.1 mm) [28] and Auscan motion analysis (10.9–22.9 mm) [26]. Study characteristics are shown in Table 3.

Selection of the superior landmark reference point varied within our included studies, with eight studies adopting C7 [13, 21, 23, 24, 26, 29–31], four studies the vertebral prominens [19, 20, 22, 27], and one study adopting T1 [28]. Similar variation was observed in the inferior reference point with two studies adopting L3 [29, 31], five studies S1 [23, 24, 26, 28, 30], five studies the midpoint between the posterior superior iliac spine (PSIS) dimples [19–22, 27], and one study adopting the superior margin of the gluteal cleft [13].

## Discussion

The aim of this systematic review was to identify, synthesise and summarise the reliability and validity of the non-radiographic global sagittal balance measurement methods. Several methods that vary widely in cost and technological complexity were identified, including plumbline testing, surface topography and IR motion analysis, which all had the most supporting evidence. Surface topography had low to moderate validity, very high reliability and high, but less than plumbline testing, accuracy. IR motion analysis had high validity and reliability with moderate accuracy. The overall quality rating of the studies was below the 60% threshold for a high rating, and they displayed a lack of homogeneity with regard to participants, reporting variables, and methods of measuring agreement.

The present systematic review noting that the plumbline method, which is the least technologically advanced and least expensive method, has high validity [21] and high reliability [29, 31]. This suggests that the plumbline method, which is easily accessible to clinicians and requires little training, can provide quantifiable data and offer higher intra-rater reliability precision than the other methods. However, a note of caution is due here as de Seze et al.’s [21] validity results were obtained from a sample of Parkinson’s disease patients exhibiting camptocormia (SVA 110 ± 11 mm), limiting generalisability to a different population.

Surface topography, unlike the other methods of measurement and with very little operator involvement, is able to provide, in one scan, the widest variety of sagittal balance measurements, including trunk inclination, distance offset measurements and sagittal arrows distance measurements. The reliability scores for inter-rater, intra-rater, inter-day and intra-day testing, including one from a high-quality study [27] ranged from high to very high reliability (ICC 0.86–0.98). However, the validity scores ranged from moderate (Pearson’s *r* of 0.68) in a low-quality study [13] to low (Pearson r of 0.49) in a high-quality study [22]. There was little consistency with regard to reporting limits of agreement of surface topography to SVA with Liljenqvist et al. [19] reporting a distance offset RMSD of 1.07 cm and Knott [22] an angular average difference of ± 3.7°. This suggests a level of inaccuracy and further work to establish clinical limits of agreement is needed, given that radiographic SVA threshold ranges defining anterior sagittal balance are 3–5 cm [6–9, 13].

Not only are our results confounded by the inconsistent selection of superior and inferior landmarks between our studies, and not all sagittal balance parameters can be measured with the same accuracy and reliability. Furthermore, the surrogate outcomes provided by non-radiographic measurement raises a question whether manually palpated surface landmarks accurately correlate with radiographic landmarks. Robinson et al. reported moderate inter-rater palpation agreement (67% within 10 mm) and moderate agreement with radiographically determined L5 (kappa 0.48) but poor agreement with radiographically determined C7 (kappa 0.18). [33]. Kilby et al. reported wide variability for manual palpation of ultrasonically identified lumbopelvic landmarks (Bland Altman limits of agreement –27 to 26 mm) concluding that manual palpation of lumbopelvic points has limited validity [34]. These validity results suggest that further research needs to be conducted to evaluate if radiographic methods of measuring global sagittal balance can be replaced with non-radiographic methods. This should be conducted with simultaneous non-radiographic evaluation of lumbar lordosis which appears to be, in conjunction with pelvic tilt, the main contributor to global sagittal balance [2, 8, 13].

The reliability of the lower cost and simpler, spinal mouse and biophotogrammetric methods, [16, 32] has been investigated to a lesser extent than plumbline, IR and surface topography. The spinal mouse system, which involves a wirelessly connected trackball, measures global sagittal balance by trunk inclination. Although validity studies are available for spinal mouse determined sagittal and coronal spine parameters, with high to very high correlation with radiographically measured coronal frontal plane Cobb angle (ICC 0.87–0.96) [35], lordosis (*r* = 0.73) and kyphosis (*r* = 0.76) angles [36], none have evaluated the validity of trunk inclination. As the spinal mouse reliability studies included in the current review involved healthy adolescent and young populations, further studies, which involve older populations need to be undertaken. In a systematic review of non-radiographic measurement of thoracic kyphosis, Barrett et al. [16] also identified strong reliability for spinal mouse measurements. Barrett et al. concluded that the flexicurve was the most feasible non-radiographic method of measuring kyphosis, with high levels of reliability and validity; however, the flexicurve cannot be used for measurement of sagittal balance.

There remains considerable debate regarding the most appropriate method of measuring agreement within reliability and validity studies [37]. Only 30% of our studies reported Bland-Altman plots, and this is less than the 85% reported in Zaki et al.’s [37] systematic review of agreement within medical instrumentation testing methods. Zaki et al. cautioned researchers about utilising inappropriate methodologies to measure agreement because they are likely to result in incorrect conclusions and possible detrimental patient care. They recommended reporting results using multiple methods of measuring agreement. The limits of agreement should also be extrapolated into clinically meaningful limits which were not detailed in any of our included studies.

### Strengths and limitations

Despite following the PRISMA guidelines, including all stages conducted by two independent reviewers, all languages and participants of any age, as with all such reviews, the possibility exists that not all the available articles were identified by the searches. We recognise that article quality may have been scored higher if the authors had adhered to the critical appraisal tool items but not reported on relevant items. We stress the importance of publication date, especially for the technology-based methods, since progressive technological evolution limits comparison of results and accuracy between and within advancing methods. There are also some limitations to be considered when interpreting our review. Due to significant variability in study methodologies, populations, reporting parameters and statistics, a quantitative meta-analysis could not be conducted.

## Conclusion

Sagittal alignment, which is associated with increased pain and reduced quality of life, is an important concept emerging within the field of spine pain and deformity care. Non-radiographic methods of measuring global sagittal balance have low to very high reliability and, limited to plumbline testing, surface topography and IR motion, low to high validity. Thus, although it is currently unclear if these three methods can be used to evaluate sagittal balance pathology, they can be used with relative confidence for the monitoring of global sagittal balance. Further research needs be undertaken to establish the value of non-radiographic methods of measuring global sagittal balance. These future studies should ideally include the ageing population, adhere to best practice research methodology and psychometric agreement statistics reporting.

## Appendix

### Medline search strategy (OVID)

1.  Physical Examination/ or ‘physical examination’.mp.
2.  exp. Kyphosis/
3.  “Sagittal balance”.mp.
4.  “Sagittal balance”.ti,ab.
5.  (Sagittal adj3 balance).mp. [mp = title, abstract, original title,
6.  imbalance*.mp.
7.  kyphosis.mp. or exp. Kyphosis/
8.  lordosis.mp. or exp. Lordosis/
9.  posture.mp. or exp. Posture/
10.  sagittal.mp.
11.  exp. Spine/ or spine.mp.
12.  exp. Cervical Vertebrae/ or “cervical spine”.mp.
13.  exp. Thoracic Vertebrae/
14.  exp. Lumbar Vertebrae/ or lumbar.mp.
15.  2 or 3 or 4 or 5 or 6 or 7 or 8 or 9 or 10 or 11 or 12 or 13 or 14
16.  reliability.mp.
17.  validity.mp.
18.  sensitivity.mp. or exp. “Sensitivity and Specificity”/
19.  responsiveness.mp.
20.  properties.mp.
21.  “inter-trial reliability”.mp.
22.  “observer variation”.mp. or exp. Observer Variation/
23.  exp. “Reproducibility of Results”/ or reproducibility.mp.
24.  16 or 17 or 18 or 19 or 20 or 21 or 22 or 23
25.  “digital photography”.mp.
26.  “flexible electrogoniometer”.mp.
27.  flexicurve.mp.
28.  kyphometer.mp.
29.  “non?invasive measurement”.mp.
30.  photogrammetry.mp. or exp. Photogrammetry/
31.  plumbline.mp.
32.  plurimeter.mp.
33.  “skin surface measures”.mp.
34.  “spinal pantograph”.mp.
35.  “spine measurement instruments”.mp.
36.  “spinal mouse”.mp.
37.  “3D scanning”.mp.
38.  “non?radiographic”.mp.
39.  25 or 26 or 27 or 28 or 29 or 30 or 31 or 32 or 33 or 34 or 35 or 36 or 37 or 38
40.  “lumbar curve”.mp.
41.  “thoracic curve”.mp.
42.  “cervical curve”.mp.
43.  “cervical curve”.mp.
44.  15 or 40 or 41 or 42
45.  15 or 40 or 41 or 42
46.  24 and 39 and 44
47.  24 and 39 and 44
48.  1 or 39
49.  24 and 44 and 48

## Abbreviations

AMED: The Allied and Complementary Medicine Database
CINAHL: The Cumulative Index of Nursing and Allied Health Literature database
DM: Midpoint between surface location of PSISs
EMBASE: The Excerpta Medica journal citation database
FP ultrasound: Freepoint ultrasound
ICC: Intra-class correlation coefficient
IR: Infra-red
ISO: International Organization for Standardization
MA: Motion analysis
MEDLINE: The National Library of Medicine journal citation database
PRISMA: The Preferred Reporting Items for Systematic Reviews and Meta-Analyses guidelines
PROSPERO: International prospective register of systematic reviews
PSIS: Posterior superior iliac spine
QUADAS: An assessment tool for the quality of diagnostic accuracy studies
QUAREL: An assessment tool for the quality of diagnostic reliability studies
*R*: Correlation coefficient
RMSD: Root mean square deviation is a measure of the difference between predicted and observed values
SEM: The standard error of measurement is the standard deviation of errors of measurement
SRS-23: Health-related quality of life questionnaire developed by the Scoliosis Research Society
SVA: Sagittal vertical axis; a measure of the horizontal offset of the midpoint of the C7 vertebrae from the posterior border of S1
T1-Spi: The angle between the midpoint of the fist thoracic vertebrae and a vertical line at the hip axis
VP: Vertabrae prominens: prominent surface location around C7, T1
